# Crystal structures of the GH18 domain of the bifunctional peroxiredoxin–chitinase CotE from *Clostridium difficile*

**DOI:** 10.1107/S2053230X20006147

**Published:** 2020-05-29

**Authors:** Jean L. Whittingham, Shumpei Hanai, James A. Brannigan, William T. Ferreira, Eleanor J. Dodson, Johan P. Turkenburg, Jared Cartwright, Simon M. Cutting, Anthony J. Wilkinson

**Affiliations:** a Structural Biology Laboratory, York Biomedical Research Institute, Department of Chemistry, University of York, York YO10 5DD, United Kingdom; b Graduate School of Pharmaceutical Sciences, Osaka University, Osaka, Japan; c School of Biological Sciences, Royal Holloway University of London, Egham, Surrey, United Kingdom; d Department of Biology, University of York, York YO10 5DD, United Kingdom

**Keywords:** *Clostridium difficile*, spores, CotE, glycosyl hydrolase, 3D domain swapping

## Abstract

CotE is a coat protein that is present in the spores of *Clostridium difficile*, an obligate anaerobic bacterium and a pathogen that is a leading cause of antibiotic-associated diarrhoea in hospital patients. Spores serve as the agents of disease transmission, and CotE has been implicated in their attachment to the gut epithelium and subsequent colonization of the host. CotE consists of an N-terminal peroxiredoxin domain and a C-terminal chitinase domain. Here, a C-terminal fragment of CotE comprising residues 349–712 has been crystallized and its structure has been determined to reveal a core eight-stranded β-barrel fold with a neighbouring subdomain containing a five-stranded β-sheet. A prominent groove running across the top of the barrel is lined by residues that are conserved in family 18 glycosyl hydrolases and which participate in catalysis. Electron density identified in the groove defines the pentapeptide Gly-Pro-Ala-Met-Lys derived from the N-terminus of the protein following proteolytic cleavage to remove an affinity-purification tag. These observations suggest the possibility of designing peptidomimetics to block *C. difficile* transmission.

## Abstract 2

*Clostridium difficile* is a spore-forming bacterium and a leading cause of hospital-acquired antibiotic-associated diarrhoea. Symptoms of disease result from secreted toxins, while disease transmission is mediated via resistant endospores. CotE is a bifunctional spore-coat protein with peroxiredoxin and chitinase domains that are implicated in colonization. Here, the structure of the chitinase domain of CotE has been determined, revealing a GH18 family fold and, unexpectedly, a peptide bound in the active site.

## 1. Introduction   

*Clostridium difficile* is an anaerobic, Gram-positive, spore-forming bacterium and an animal pathogen which has emerged as the most frequent cause of antibiotic-associated hospital-acquired diarrhoea (Smits *et al.*, 2016 ▸). It is estimated that there are half a million new cases each year of infection with *C. difficile* (CDI), augmented by 75 000–175 000 cases of recurrent CDI. Treatment with antibiotics such as fidaxomicin cures 90% of new cases of CDI; however, recurrent infection is common and thousands of patients are on costly long-term antibiotic treatment, with more than 25 000 deaths per annum in the USA alone (Lessa *et al.*, 2015 ▸). The disease follows antibiotic therapy, which disrupts the normal gut microflora, providing an opportunity for *C. difficile* colonization. The pathogen causes a range of clinical conditions in humans which range from mild diarrhoea to life-threatening pseudomembranous colitis, toxic megacolon and colonic perforation.

Spores play an important role in CDI as the agents of disease transmission. As robust and metabolically dormant structures, *C. difficile* spores can survive the dysbiosis of the gut microflora induced by antibiotic treatment. The resulting perturbation of the intestinal microbiota creates an environment in which the spore can germinate and proliferate as vegetatively growing cells, leading to colonization of the gastrointestinal tract. Disease is primarily associated with the secretion of two inflammatory cytotoxins, TcdA and TcdB, which are responsible for tissue damage (Shen, 2012 ▸). These large toxins enter the cells of the colonic epithelium, where they glucosylate small GTPases, disrupting signalling in these cells and giving rise to cytopathic effects and cytoxicity. Toxin synthesis and spore formation by *C. difficile* may take place simultaneously upon nutrient limitation, so that while non­sporulating cells in the population produce toxins to generate nutrients, the sporulating cells are designed for survival, dissemination and the initiation of new infections (Daou *et al.*, 2019 ▸).

The spore has an elaborate multi-layered structure. Analysis of the proteins released from spores of *C. difficile* following treatment with SDS–borate–dithiothreitol identified a number of spore-coat enzymes, including superoxide dismutase and catalase (Permpoonpattana *et al.*, 2011 ▸). Among the most interesting of the discovered proteins was CotE, an 81 kDa protein comprising an N-terminal domain homologous to 1-Cys peroxiredoxins, a central cysteine-rich interdomain region and a C-terminal domain with homology to family 18 glycosyl hydrolases (GH18). The corresponding enzyme activities were demonstrated for purified recombinant polypeptides comprising these domains (Permpoonpattana *et al.*, 2013 ▸). The roles of these enzymatic activities in the spore are intriguing. Insertional mutagenesis showed that *cotE* is not essential for spore integrity (Permpoonpattana *et al.*, 2013 ▸); however, in animal models of CDI, when CotE is absent the capacity of spores to colonize the intestine and induce virulence is markedly reduced (Hong *et al.*, 2017 ▸). It was proposed that CotE facilitates host colonization by binding to the mucus layer of the intestine. This in turn implies that CotE may be a target for the prevention of or intervention in CDI. To build a greater understanding of structure–function relationships and to provide a platform for future inhibitor discovery, we have determined crystal structures of the chitinase domain of CotE.

## 2. Materials and methods   

### 2.1. Macromolecule production   

#### 2.1.1. Cloning   

The oligonucleotide primers CotE-349F and CotE-712R (Table 1 ▸) were used to amplify a 1119 bp fragment from a pET-28b plasmid derivative harbouring a *cotE* sequence codon-optimized for expression in *Escherichia coli*. The fragment produced by polymerase chain reaction (PCR) was inserted into the plasmid pETYSBLLIC3C (Fogg & Wilkinson, 2008 ▸) by the In-Fusion method (Clontech Laboratories) and the products were used to transform *E. coli* strain XL1-Blue. Plasmids from kanamycin-resistant colonies were prepared and their ‘cloned’ DNA inserts were sequenced to confirm the presence and authenticity of the expected inserts. The plasmid pETYSBLLIC3C-CotEC was used to transform *E. coli* BL21 (DE3) cells. It encodes residues 349–712 of CotE fused N-terminally to the sequence MGSSHHHHHHSSGLEVLFQGPA comprising a human rhinovirus (HRV) 3C-cleavable hexahistidine tag (Table 1 ▸).

#### 2.1.2. Protein purification   

Recombinant protein was produced in *E. coli* BL21(DE3) cells. The cells were grown with shaking at 310 K in Luria–Bertani broth containing 30 mg ml^−1^ kanamycin to an OD_600_ of 0.6–0.8. Recombinant protein production was induced by the addition of 1 m*M* isopropyl β-d-1-thiogalactopyranoside and incubation at 289 K for 20 h. The cells were harvested by centrifugation, the pellet was resuspended in buffer *A* (50 m*M* Tris–HCl pH 7.5, 500 m*M* NaCl, 10 m*M* imidazole), to which an EDTA-free protease-inhibitor cocktail tablet (Roche Diagnostics, USA) had been added, and the cells were lysed by sonication on ice. The soluble cell extract was collected following centrifugation and CotE(349–712) was purified in three steps, each of which was carried out at room temperature. Firstly, the soluble cell supernatant was loaded onto a 5 ml nickel-charged HisTrap column (Amersham Pharmacia) equilibrated in buffer *A*. After washing, the column was developed with a 10–500 m*M* imidazole gradient in buffer *A*. Fractions containing CotE(349–712) were identified and pooled. The pooled sample (∼2.5 mg ml^−1^ protein concentration) was treated with a 1:50 ratio of HRV 3C protease (purified in-house) to cleave off the N-terminal tag with simultaneous dialysis overnight against buffer *B* (20 m*M* Tris–HCl pH 7.5, 150 m*M* NaCl). The cleavage products were passed over a second Ni–NTA agarose column equilibrated in buffer *B*. In this step, highly purified untagged protein was identified in the flowthrough fractions, which were combined, concentrated by centrifugal ultrafiltration (Amicon Ultra) and passed through a Superdex S200 column in buffer *B*. After gel filtration, the molecular mass of the purified protein was measured by electrospray ionization mass spectrometry to be 17 339 Da, which is within 1 Da of the calculated mass of CotE(349–712) with an N-terminal Gly-Pro-Ala sequence representing a vestige of the cloning and proteolysis procedure.

### 2.2. Crystallization   

Protein concentrations were determined with an Epoch Microplate Spectrophotometer using an extinction coefficient at 280 nm calculated from the sequence. Crystallization experiments were set up as sitting drops in 96-well plates using Hydra 96 and Mosquito liquid-handling systems to dispense the reservoir and drop solutions, respectively. Drops consisting of 150 nl 20 mg ml^−1^ protein solution in 20 m*M* Tris–HCl pH 8.0, 150 m*M* NaCl and 150 nl reservoir solution were equilibrated against 0.1 ml reservoir solution. Crystals of CotE(349–712) were obtained following screening experiments with Clear Strategy Screens I and II (Brzozowski & Walton, 2001 ▸) under conditions that contained PEG 3350. Refinement of these conditions in a hanging-drop format led to the growth of well diffracting crystals from drops that were formed by mixing 1 µl protein solution with 2 µl reservoir solution composed of 200 m*M* ammonium phosphate, 22.5% PEG 3350 and were equilibrated against 1 ml reservoir solution (Table 2 ▸). These crystals belonged to space group *P*2_1_ and are referred to as the monoclinic crystal form.

Following the discovery of electron density in the putative active-site region, we sought to remove the co-purifying ligand. For this purpose, we immobilized the His_6_-tagged CotE(349–712) on a Ni^2+^-chelation column in buffer *A* and washed the column extensively with buffer *A* containing 2 *M* guanidine hydrochloride followed by a 2–0 *M* descending gradient of guanidine hydrochloride in buffer *A*. The polyhistidine tag was subsequently removed from the protein as described above. Crystallization experiments produced diffracting crystals from hanging drops formed by mixing 2 µl 20 mg ml^−1^ CotE(349–712) with 3 µl well solution consisting of 1 ml 0.1 *M* sodium malonate pH 5.5, 13% PEG 3350 and 3 µl pentaethylene glycol monooctyl ether (C_8_E_5_) (Table 2 ▸). These crystals belonged to space group *P*6_1_22 and are referred to as the hexagonal crystal form.

### 2.3. Data collection and processing   

Single crystals of CotE(349–712) were captured from crystallization drops in fine nylon loops and transferred to a solution of mother liquor containing 15%(*v*/*v*) glycerol prior to cryocooling in liquid nitrogen. Crystals were tested on an in-house system and the best diffracting crystals were chosen and sent to Diamond Light Source (DLS) for data collection to high resolution. For the monoclinic and hexagonal crystal forms, data were collected on beamlines I02 and I03, respectivley. The data were processed using the 3dii option in *xia*2 (Winter, 2010 ▸) and extended to 1.3 and 2.2 Å resolution, respectively, for the monoclinic and hexagonal crystal forms (Table 3 ▸). For both crystal forms, there is one protein molecule per asymmetric unit.

### 2.4. Structure solution and refinement   

The structures of CotE(349–712) were determined and refined using the *CCP*4 suite of software as implemented in the *CCP*4*i*2 graphical interface (Potterton *et al.*, 2018 ▸). The structure of the monoclinic crystal form was solved by molecular replacement in *Phaser* (McCoy *et al.*, 2007 ▸) using the coordinate set for the catalytic domain of chitinase A1 (ChiA1) from *Bacillus circulans* (PDB entry 1itx; Matsumoto *et al.*, 1999 ▸) as the search model. The proteins share 35% sequence identity across 353 and 468 aligned residues of CotE and ChiA1, respectively. The resulting model was refined in *REFMAC*5 (Murshudov *et al.*, 2011 ▸) and automatic model building was then performed using *Buccaneer* (Cowtan, 2006 ▸). This was followed by iterative rounds of manual model building in *Coot* (Emsley *et al.*, 2010 ▸) and refinement in *REFMAC*5. The refinement statistics are summarized in Table 4 ▸. The structure of the hexagonal crystal form was solved by molecular replacement using the refined coordinates of the structure of the monoclinic form.

## 3. Results and discussion   

### 3.1. Determination of the structure of the CotE chitinase domain   

We initiated structural studies of CotE by generating a series of expression constructs encoding C-terminal fragments of different lengths fused to a cleavable polyhistidine tag. This allowed us to identify CotE(349–712) as a stable, soluble fragment. The fragment encompasses the chitinase domain, which is predicted to span residues 380–685. This protein was purified from overproducing *E. coli* by immobilized nickel-affinity chromatography, cleavage to remove the affinity tag and gel-filtration chromatography. The protein was first crystallized from polyethylene glycol 3350-containing solutions. The crystals belonged to space group *P*2_1_, with a single protein molecule in the asymmetric unit and a solvent content of 48%. Data extending to 1.3 Å resolution were collected at the Diamond Light Source synchrotron-radiation source and the structure was solved by molecular replacement using the coordinates of the catalytic domain of chitinase A1 from *B. circulans* (PDB entry 1itx; Matsumoto *et al.*, 1999 ▸) as the search model. The CotE(349–712) structure has been refined to give a crystallographic *R* value of 10.6% (*R*_free_ = 13.3%) for a model comprising residues Ile363–Phe712, 490 waters and a pentapeptide-like entity (currently modelled as Gly-Pro-Ala-Met-Lys) defined by residual electron density located in the active-site region of the structure.

### 3.2. Structure description   

**Figure 1:**
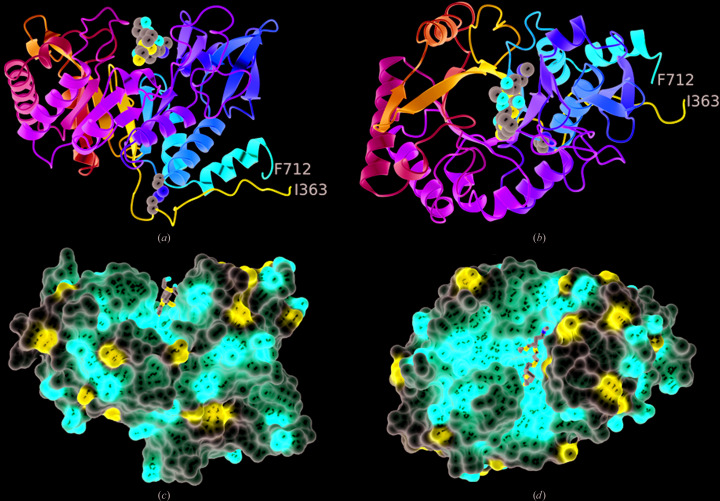
The overall structure of the chitinase domain of CotE. (*a*, *b*) Ribbon rendering of the polypeptide chain colour-ramped from the N-terminus (Ile363, blue) to the C-terminus (Phe712, red). The atoms of the disulfide bond linking cysteines 376 and 670 are shown as spheres, as are the atoms of the pentapetide ligand located in the active site. These atoms are coloured by element, with C in grey, O in red, N in blue and S in yellow. The views are from the side of the β-barrel (*a*) and looking down into it (*b*). (*c*, *d*) Electrostatic surface renderings of the protein in similar orientations to those in (*a*) and (*b*), respectively. The prominent groove that forms the active site and its markedly negative electrostatic potential are apparent.

CotE(349–712) has a classical parallel eight-stranded αβ-barrel at its core, on top of which the substrate-binding site resides (Fig. 1 ▸). The (αβ)_8_-barrel is compact, with the exception of significant insertions following strands β6 and β7. Following β7, residues 605–655 form a distinct subdomain comprising a five-stranded β-sheet and an α-helix which packs across one face of this sheet. The opposite face of the sheet packs against the meandering segment of the polypeptide following strand β6, which spans residues 550–580. Much of the structure is covalently closed through a disulfide bond linking residues Cys376 and Cys670 (Fig. 1 ▸).

A search of the Protein Data Bank for structures similar to the CotE chitinase domain revealed a plethora of coordinate sets with *Q*-scores greater than 0.5, corresponding to r.m.s.d. values in the range 1.3–1.4 Å over 300 or so C^α^ atoms of matched residues. These included human chitinase/chitotriosidase (PDB entry 1lg2; Fusetti *et al.*, 2002 ▸), the mouse lectin YM1 (PDB entry 1vf8; Tsai *et al.*, 2004 ▸) and bacterial chitinases from *Serratia proteamaculans* (PDB entry 4lgx; Madhuprakash *et al.*, 2015 ▸) and *Klebsiella pneumonia* (PDB entry 3qok; Midwest Center for Structural Genomics, unpublished work) variously in complexes with monosaccharides, disaccharides and oligosaccharides as well as oligosaccharide and peptide-based inhibitors.

### 3.3. The substrate-binding and active site   

**Figure 2:**
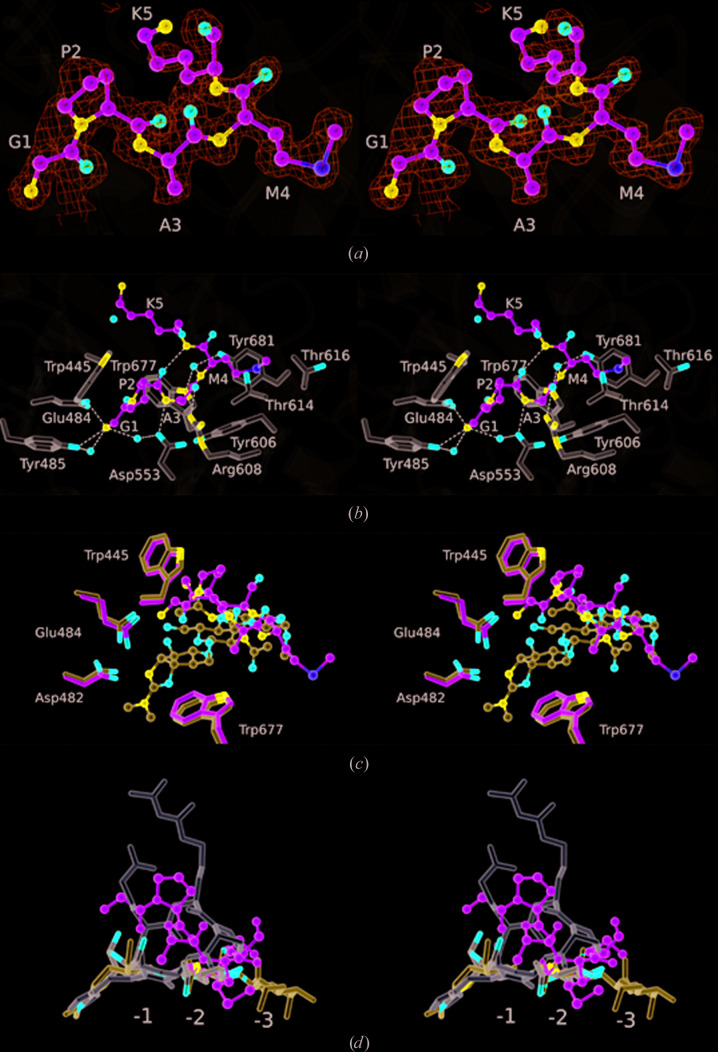
Peptide–CotE interactions. Stereo images are shown with the pentapeptide ligand in ball-and-stick format. (*a*) The peptide with its associated electron density in maps calculated with coefficients 2*mF*_o_ − *DF*_c_ and contoured at 1σ. Residues are labelled using the single-letter code. (*b*) The pentapeptide ligand displayed as above, with surrounding protein residues in cylinder format (with three-letter code labels). Atoms are coloured by element type (O, red; N, blue; S, yellow; C in green for the ligand or light grey for the protein). Neighbouring water molecules are shown as red spheres. Polar interactions are denoted by dashed lines. (*c*) Superposition of the methylallosamidin ligand (blue C atoms) from the complex with human chitinase (PDB entry 1hkj; Rao *et al.*, 2003 ▸) and the GPAMK ligand (green C atoms) from CotE. Selected conserved residues are shown and numbered as in CotE. The structures were displayed in *CCP*4*mg* (McNicholas *et al.*, 2011 ▸) following *SSM* superposition, which gave a r.m.s.d. of 1.4 Å for 292 matching atoms. (*d*) The argadin (white; PDB entry 1waw; Rao *et al.*, 2005 ▸), methylallosamidin (blue; PDB entry 1hkj; Rao *et al.*, 2003 ▸) and chitobiose (coloured by atoms with C in grey and O in red; PDB entry 4wkh; Fadel *et al.*, 2015 ▸) ligands from human chitinase structures superposed on the GPAMK ligand from CotE. The binding subsites −3, −2 and −1 are labelled.

A pronounced groove runs across the top of the molecule when viewed in the orientation shown in Fig. 1 ▸(*c*). In family GH18 enzymes, this forms the substrate-binding and active site. As seen in the structures of other GH18 family members, this groove has a markedly negative electrostatic potential (Figs. 1 ▸ *c* and 1 ▸ *d*). Located in this groove, we observed a strong positive feature in the electron-density maps that we were able to model as a pentapeptide: Gly-Pro-Ala-Met-Lys (Fig. 2 ▸ *a*). There is uncertainty for the side chain of residue 5, but the sequence otherwise corresponds to the first five residues of the expected product of HRV 3C protease cleavage of the recombinant fusion protein. The Gly-Pro-Ala segment is a vestige of the cleavage-recognition sequence, with the Met-Lys segment constituting residues 349 and 350 of CotE. The binding site for the peptide is circumscribed by residues Met550, Tyr485, Glu484, Trp445, Asp553, Ala556, Arg608, Thr614, Thr616, Tyr606, Tyr681 and Trp677, forming a protein–peptide interface of 362 Å^2^ (Fig. 2 ▸ *b*).

The α-amino group of the peptide makes an ion-pairing interaction with the side-chain carboxylate of Glu484 and a charge–dipole interaction with the phenolic hydroxyl group of Tyr485, as well as polar interactions with surrounding water molecules, one of which forms a bridging polar interaction with the carboxylate of Asp553 (Fig. 2 ▸ *b*). The face of the pyrrolidine ring of Pro2 and the residue 1–2 peptide bond pack closely with the face of the indole ring of Trp445. The carbonyl O atom of Pro2 makes an intramolecular hydrogen bond to the amide >N–H of Lys5, while the amide >N–H of Ala3 makes a charge–dipole interaction with the side-chain carboxylate of Asp553 (Fig. 2 ▸ *b*). Further along the ligand backbone, the carbonyl O atom of Ala3 forms another charge–dipole interaction with the guanidino group of Arg608. The backbone >N—H of Met4 forms a hydrogen bond to a well ordered water molecule, with bridging hydrogen bonds to the indole N atom of Trp677 and the phenolic hydroxy group of Tyr681. The side chain of Met4 projects into a pocket lined by the side chains of the protein residues Tyr606 and Tyr681 and the aliphatic faces of Thr614 and Thr616 (Fig. 2 ▸ *b*). The electron density becomes more diffuse at Lys5, precluding further model building.

The peptide residing in the binding groove may be an N-terminal degradation product of proteolysis. This would imply high-affinity binding, since the peptide is evidently retained on passage through a gel-filtration column. Alternatively, this pentapeptide may represent the amino-terminus of the intact CotE chitinase-domain polypeptide. If this is the case, then the intervening residues Thr351–Thr362, which are not visible in the electron-density maps, are missing owing to disorder. Lys5 of the peptide and Ile363, the first residue of the polypeptide defined by the electron-density maps, are on opposite faces of the chitinase domain (Fig. 1 ▸ *a*) and 45 Å apart, so it is unlikely that they belong to the same molecule. The same lysine and Ile363 in the protein molecule generated by the symmetry operation (−*x*, *y* − 1/2, −*z*) are separated by 24 Å, a distance that could be spanned by the 12 ‘missing’ residues.

### 3.4. Mechanistic considerations   

Chitinases are enzymes that catalyse glycosidic bond cleavage in β-1,4-linked *N*-acetylglucosamine (GlcNAc)-containing polymers such as chitin. Chitin is widely distributed in nature, but the physiological substrate of CotE is not known. Polymeric structures containing GlcNAc are present in the cell wall of *C. difficile*, a structure that is known to undergo remodelling during spore formation. The integrity of *C. difficile* spores, however, is unaffected by deletion of *cotE*, suggesting that the substrate may be host-derived. Chitin does not occur in mammals, suggesting that the target of CotE action during infection is a glycoprotein, and evidence has been presented to show that CotE facilitates spore binding to mucin and mucin degradation (Hong *et al.*, 2017 ▸).

The family 18 glycosyl hydrolases carry a conserved acidic motif, which occurs in CotE as D^477^GIDIDWEY^485^. As shown in Fig. 2 ▸(*b*), Glu484 and Tyr485 form polar contacts to the α-amino group of the bound peptide in CotE. The reaction mechanisms of these enzymes feature neighbouring-group assistance, in which the carbonyl of the acetyl group on the GlcNAc in the −1 site acts as the nucleophile (Terwisscha van Scheltinga *et al.*, 1995 ▸; Tews *et al.*, 1997 ▸). An Asp residue (corresponding to Asp482 in CotE) stabilizes the developing positive charge on the –NH of the acetyl group, while a glutamic acid (corresponding to Glu484) promotes breakage of the β-1,4 glycosidic linkage between residues bound in the −1 and +1 subsites by protonating the leaving group. This results in the formation of an oxazolinium intermediate in the −1 site, the positive charge of which is stabilized by neighbouring carboxylates. A water molecule, activated by the Glu residue now serving as a base, attacks at the anomeric C atom with opening of the oxazolinium ring and reformation of the *N*-acetyl group. As a result, there is an overall retention of configuration at the anomeric C atom (Terwisscha van Scheltinga *et al.*, 1995 ▸).

There has been much research into chitinase inhibition, since chitinase inhibitors have potential applications in the treatment of human diseases, including those resulting from bacterial infections (Frederiksen *et al.*, 2013 ▸). As a result, many classes of chitinase inhibitor have been discovered, including sugar derivatives such as the natural product allosamidin and derivatives thereof (Sakuda *et al.*, 1986 ▸; Mac­­donald *et al.*, 2010 ▸). The structure of the CotE–peptide complex is compared with that of human macrophage chitinase bound to methylallosamidin (Rao *et al.*, 2003 ▸) in Fig. 2 ▸(*c*). Following structure superposition by the *SSM* procedure as implemented in *CCP*4*mg* (McNicholas *et al.*, 2011 ▸), the r.m.s.d. for 292 matching atoms is 1.4 Å. Methylallosamidin, which consists of two β-1,4-linked *N*-acetylglucosamine residues attached to allosamizoline, binds in the −3 to −1 subsites in the chitinase substrate-binding site. Allosamizoline is bicyclic, consisting of a cyclopentitol ring fused to an oxazoline, which mimics the oxazolinium intermediate in the chitinase-catalysed reaction. As seen in Fig. 2 ▸(*c*), the peptide-binding site in CotE partially overlaps with the allosamidin-binding site in human chitinase, particularly in the −3 and −2 subsites.

The cyclic pentapeptides argifin and argadin are of interest in relation to the peptide bound in the substrate-binding site of the chitinase domain of CotE. Argifin and argadin are produced by fungi and have a high potency (nanomolar) towards their insect chitinase targets (Shiomi *et al.*, 2000 ▸). Structures have been determined of these inhibitors in complex with bacterial and human chitinases, revealing mimicry of the natural carbohydrate substrate (Rao *et al.*, 2005 ▸). Relative to the GPAMK peptide bound to CotE, the cyclic peptide argadin binds more deeply in the substrate-binding cleft. In Fig. 2 ▸(*d*), the argadin (white), methylallo­samidin (blue) and chitobiose (grey) ligands from human chitinase complexes are overlaid on the GPAMK peptide (green) from the CotE complex. Argadin occupies subsites −2 and −1, defined by the binding of the chitobiose (GlcNAc_2_), and extends towards the +1 site to make interactions with the catalytic residues (Fadel *et al.*, 2015 ▸). In its complex, the argadin conformation is stabilized by extensive intramolecular polar interactions. In contrast, there are few polar interactions with the protein; instead, there is quite an extensive buried surface area which will contribute to higher affinity.

### 3.5. Domain swapping   

Before we confirmed the identity of the peptide ligand in the chitinase-domain substrate-binding groove, we sought to remove this ‘ligand’ and crystallize the protein in an un­liganded form. To obtain unliganded CotE(349–712), the first Ni–NTA column-chromatography purification procedure was modified so that after the binding and washing steps, the column was washed with buffer *C* (buffer *A* containing 2 *M* guanidine hydrochloride) to partially unfold the immobilized protein and allow the dissociation of endogenous ligand(s). We have used this technique previously to remove ligands from peptide-binding proteins (Hughes *et al.*, 2019 ▸). After washing with ten column volumes of buffer *C*, the concentration of guanidine hydrochloride was decreased to zero in a series of five steps. The protein was then eluted from the column by applying an increasing imidazole concentration gradient. The eluted recombinant protein was subsequently cleaved with HRV 3C protease and further purified as before.

**Figure 3:**
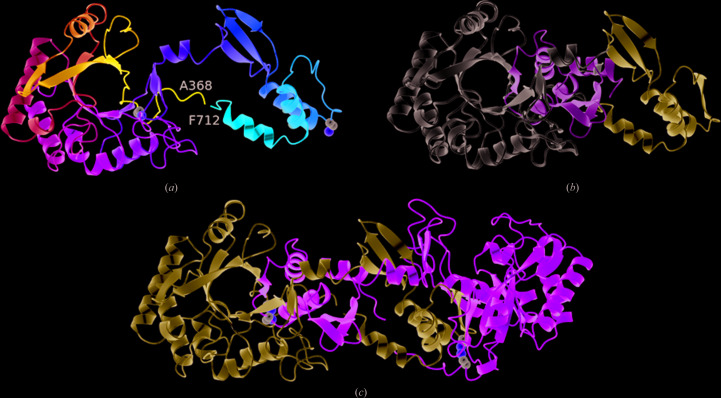
Domain swapping in the chitinase domain of CotE. (*a*) Ribbon rendering of the chitinase domain in the *P*6_1_22 crystal structure. The chain is colour-ramped as in Fig. 1 ▸(*a*), and the C^α^ atoms and side chains of cysteines 376 and 670 are shown as spheres. The C-terminal residues 627–712 extend away from the β-barrel so as to pack onto and complete the β-barrel of a crystallographic symmmetry mate, as shown in (*c*), where the two subunits of the domain-swapped dimer are coloured light blue and green, respectively. The eighth strand of each β-barrel is provided by the partner subunit. (*b*) A juxtaposition of the C-terminal swapped domains (residues 627–712) in the *P*2_1_ and *P*6_1_22 crystal forms, coloured light green and ice blue, respectively, relative to the β-­barrel domain (in white) following the superposition of residues 358–623 is shown.

In retrospect this strategy was flawed, since the peptide ligand GPAMK is presumably generated during or after the HRV 3C protease-cleavage step. The partially unfolded/refolded protein was crystallized in a different crystal form (*P*6_1_22). Solution of the structure and refinement against data extending to ∼2.1 Å resolution revealed that the substrate-binding groove was indeed empty. However, the structure proved to be of a 3D domain-swapped dimer. In this structure (Fig. 3 ▸), domain exchange takes place at residue Lys627. As is usual following 3D domain swapping, the respective domains in the two structures can be closely superposed. Thus, 244 C^α^ atoms in the residue range 368–623 can be superposed with an r.m.s.d. of 0.44 Å, while similarly 78 C^α^ atoms of residues 627–708 can be superposed with an r.m.s.d. of 0.43 Å.

3D domain swapping is commonly observed in protein crystal structures since it is promoted by high protein concentrations and/or partially denaturing conditions (Schlunegger *et al.*, 1997 ▸). Indeed, we have observed this phenomenon in crystals of other sporulation proteins. In Spo0A from *B. subtilis* (Lewis *et al.*, 2000 ▸) and CodY from *C. difficile* (Daou *et al.*, 2019 ▸) α-helices are swapped, leading to dimeric and hexameric assemblies, respectively, while in SpoIIE from *B. subtilis* (Levdikov *et al.*, 2012 ▸) three β-strands from a β-sandwich are exchanged in a 3D domain-swapped dimer. In almost all instances 3D domain swapping is a crystallographic artefact and there is no evidence that the 3D domain-swapped dimer of CotE(349–712) is physiologically significant. It is nevertheless structurally interesting since it involves (i) the exchange of a strand, β8, from the β-barrel and (ii) the breakage of the intramolecular disulfide bond between Cys376 and Cys670 and the formation of an intermolecular equivalent.

3D domain swapping in the TIM barrel of a subunit of tryptophan synthase (TrpA) from *Streptococcus pneumoniae* was recently observed (Michalska *et al.*, 2020 ▸). In TrpA, the N-terminal strands 1 and 2 of the TIM barrel are exchanged in dimer formation. In this paper, the authors surveyed 2000 or so TIM-barrel structures in the PDB and found that the integrity of the core barrel was always preserved. They concluded that their TrpA structure is the first example of 3D domain swapping that intrudes into the core of the barrel (Michalska *et al.*, 2020 ▸). The 3D domain-swapped CotE(349–712) structure reported here is therefore the second example, with the distinction that it is the C-terminal strand 8 of the barrel that is swapped.

## Supplementary Material

PDB reference: chitinase domain of CotE, hexagonal crystal form, 6tsb

PDB reference: monoclinic crystal form, 6t9m

## Figures and Tables

**Table 1:** Macromolecule-production information

Source organism	*C. difficile*
DNA source	Synthetic codon-optimized sequence
Forward primer CotE-349F	5′-TCCAGGGACCAGCAATGAAAACCCTGAAAGATAGC-3′
Reverse primer CotE-712R	5′-TGAGGAGAAGGCGCGTTAAAACTGGCCATAAATACCTTCC-3′
Cloning vector	pETYSBLLIC3C
Expression vector	pETYSBLLIC3C
Expression host	*E. coli* BL21(DE3)
Complete amino-acid sequence of the construct produced	MGSSHHHHHHSSGLEVLFQGPAMKTLKDSKKLVRPQITDPYNPIVENANCPDINPIVAEYVLGNPTNVDAQLLDAVIFAFAEIDQSGNLFIPYPRFLNQLLALKGEKPSLKVIVAIGGWGAEGFSDAALTPTSRYNFARQVNQMINEYALDGIDIDWEYPGSSASGITSRPQDRENFTLLLTAIRDVIGDDKWLSVAGTGDRGYINSSAEIDKIAPIIDYFNLMSYDFTAGETGPNGRKHQANLFDSDLSLPGYSVDAMVRNLENAGMPSEKILLGIPFYGRLGATITRTYDELRRDYINKNGYEYRFDNTAQVPYLVKDGDFAMSYDDALSIFLKTQYVLRNCLGGVFSWTSTYDQANILARTMSIGINDPEVLKEELEGIYGQF

**Table 2:** Crystallization

Crystal	CotE(349–712), monoclinic	CotE(349–712), hexagonal
Method	Vapour diffusion in hanging drops	Vapour diffusion in hanging drops
Plate type	24-well	24-well
Temperature (K)	291	291
Protein concentration (mg ml^−1^)	20	20
Buffer composition of protein solution	20 m*M* Tris–HCl pH 8.0, 150 m*M* NaCl	50 m*M* Tris–HCl pH 7.5, 500 m*M* NaCl, 10 m*M* imidazole
Composition of reservoir solution	200 m*M* ammonium phosphate, 22.5% PEG 3350	1 ml 0.1 *M* sodium malonate pH 5.5, 13% PEG 3350 and 3 µl C_8_E_5_
Volume and ratio of drop	3 µl (1 µl protein solution + 2 µl reservoir solution)	5 µl (2 µl protein solution + 3 µl reservoir solution)
Volume of reservoir (ml)	1	1

**Table 3:** Data collection and processing Values in parentheses are for the outer shell.

Crystal	CotE(349–712), monoclinic	CotE(349–712), hexagonal
Diffraction source	I02, DLS	I03, DLS
Wavelength (Å)	0.9795	0.9763
Temperature (K)	100	100
Detector	Dectris PILATUS3 6M-F	Dectris PILATUS3 6M
Crystal-to-detector distance (mm)	218.05	390.17
Rotation range per image (°)	0.1	0.1
Total rotation range (°)	250	180
Exposure time per image (s)	0.04	0.04
Space group	*P*2_1_	*P*6_1_22
*a*, *b*, *c* (Å)	45.9, 54.9, 80.3	82.1, 82.1, 325.9
α, β, γ (°)	90, 101.4, 90	90, 90, 120
Resolution range (Å)	26.3–1.30 (1.32–1.30)	81.41–2.17 (2.23–2.17)
Total No. of reflections	434288 (21619)	654223 (47083)
No. of unique reflections	94345 (4564)	35657 (2551)
Completeness (%)	97.9 (96.1)	100 (100)
Multiplicity	4.6 (4.7)	18.3 (18.5)
〈*I*/σ(*I*)〉	18.0 (6.5)	9.5 (1.5)†
*R*_meas_	0.058 (0.31)	0.19 (2.43)
CC_1/2_	0.997 (0.954)	1.0 (0.64)
Overall *B* factor from Wilson plot (Å^2^)	9.3	39.1

† The mean *I*/σ(*I*) falls below 2 at a resolution of 2.2 Å. The data yielded valuable information as shown by CC_1/2_.

**Table 4:** Structure refinement Values in parentheses are for the outer shell.

Crystal	CotE(349–712), monoclinic	CotE(349–712), hexagonal
Resolution range (Å)	45.09–1.30 (1.33–1.30)	54.37–2.10 (2.15–2.10)
Completeness (%)	97.89 (96.11)	99.96 (99.93)
σ Cutoff	None	None
No. of reflections		
Working set	89793 (6466)	37274 (2679)
Test set	4557 (328)	1918 (154)
Final *R*_cryst_	0.106 (0.119)	0.22 (0.41)
Final *R*_free_	0.133 (0.158)	0.27 (0.43)
Cruickshank DPI	0.039	
No. of non-H atoms		
Protein	2843	2699
Additives	53	14
Ligand	33	—
Water	490	138
Total	3419	2851
R.m.s. deviations		
Bonds (Å)	0.020	0.016
Angles (°)	2.47	1.78
Average *B* factors (Å^2^)		
Protein	13.5	58.8
Additives	33.0	90.3
Ligand	28.2	—
Water	29.6	64.0
Ramachandran plot		
Favoured regions (%)	90.1	87.5
Additionally allowed (%)	9.9	11.8
Outliers (%)	0	0.3
